# Supplementary material for: Detection of Antibodies against Endemic and SARS-CoV-2 Coronaviruses with Short Peptide Epitopes
Source: Vaccines (Basel). 2023 Aug 23;11(9):1403. doi: 10.3390/vaccines11091403 (PMC10535424; doi:10.3390/vaccines11091403)
Supplement: Supplementary file 1 [file vaccines-11-01403-s001.zip › 230807_suppl_vaccines-2532610.pdf]

Supplement

S1. Technical description of phage display, NGS and final peptide selection

Selection procedure: The procedure started with enrichment of the binding peptides. Two rounds of selection were performed. Serum antibodies, without prior purification, were bound to protein A beads before adding the phage library. Antibodies in the serum could bind to the peptides displayed on the phage particles and the unbound phage particles were washed away. The remaining phage particles used for re-amplification in E. coli. The DNA from phage particles of both selection rounds was prepared for NGS and used for further analysis. The NGS data were processed using PEAR and Trimmomatic software. Processed datasets were analyzed with special software called "LibDB."

Statistical Analysis: LibDB displays the number of all sequences present in the dataset of one serum. Statistical analysis was conducted for 3-mer and 4-mer motifs of sequences, whereby three parameters were calculated: relative frequency, expected value according to the theoretical ENTE-1 design, and enrichment of the respective motif.

Epitope mapping: The enrichment of 4-mer motif statistics in all serum datasets of interest can be subsequently depicted in a diagram for the examined protein antigen. The 4-mer motifs of the antigen sequence are displayed on the x-axis and the enrichment is shown on the y-axis. The resulting diagram can be used to identify the motifs of potential epitopes. Identified sequences sharing a motif were aligned, and potential epitopes were matched with the 3D-protein structure. Peptides containing epitope sequences were synthesized and tested using a peptide microarray.

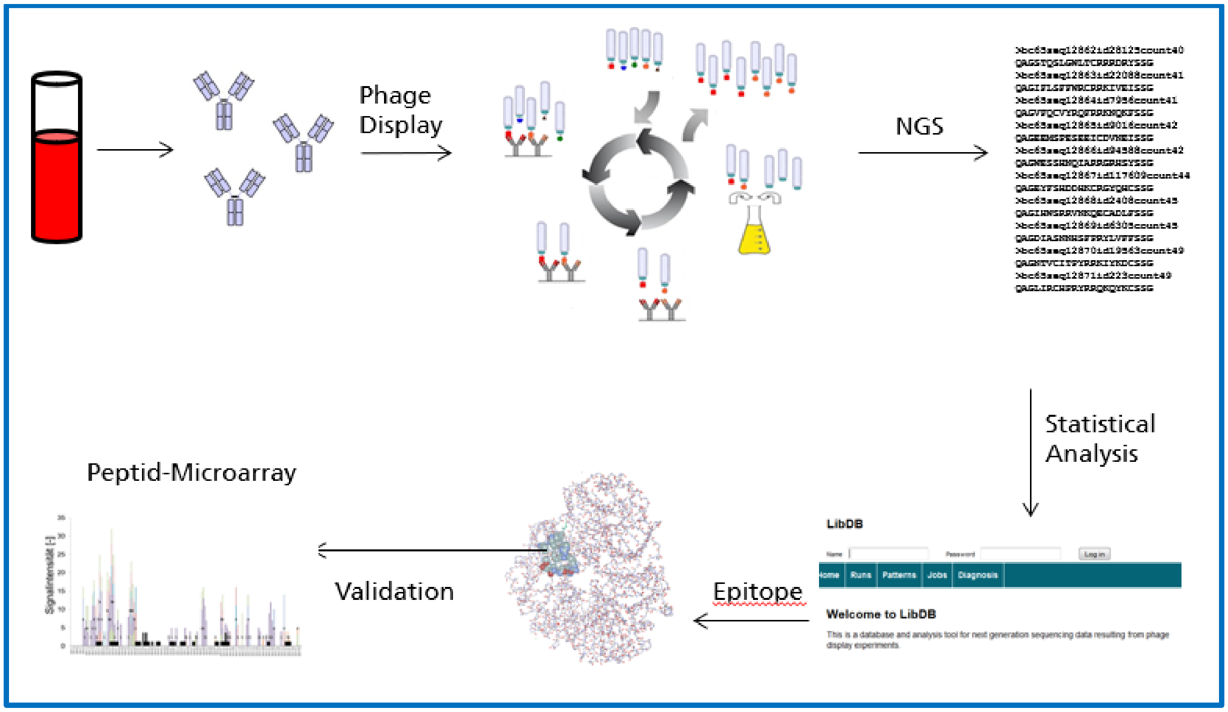

Figure S1-1: Overview of the processes used for epitope mapping.

S2. Detailed explanation of array data analyses

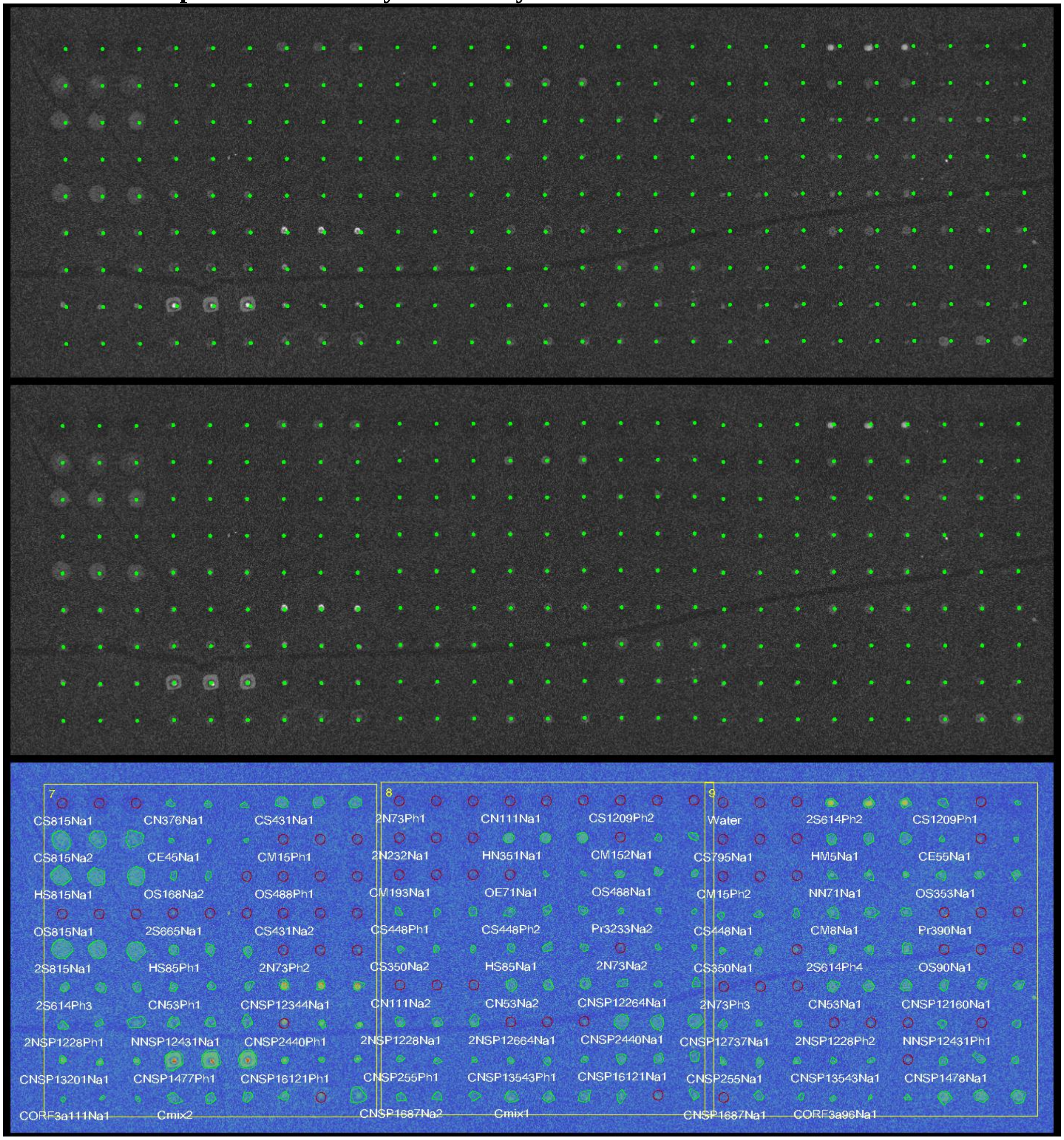

Figure S2-1: This figure shows the three main steps during the image processing of the microarrays in this project from top to bottom. All three pictures show the same section of three blocks out of a larger array image with 24 blocks, which had been gamma-transformed before to amplify lower intensities. The image at the top is the result of grid detection with a correlation technique that uses the entire grid at once. Therefore, it does not focus on single spot or block inaccuracies, but rather attempts to find a compromise for the entire grid of 24 blocks. This is seen by observing the rightmost spots in the top image, which are slightly offset to the left, compared to the aligned green positions. This is corrected in the next step (center image), where another alignment step takes place for only one block at a time. After this step, the detected positions do not have to fit perfectly in the center of each spot because the small spot-specific inaccuracies are compensated by the segmentation algorithm, which uses a geodesic dilation-based thresholding algorithm to extract predominantly convex spot shapes. The resulting spot segments were displayed to the user with a rainbow color scale and associated labels for convenient validation (bottom image) before further analysis of the data in a spreadsheet format was performed.

S3. Alignments of peptide epitopes with antigen sequences

Sequence alignments of the peptides used in the peptide array (Fig. 2) with different corona virus proteins. If possible, the alignments have been carried out with both SARS proteins, the endemic strain and another strain recently described. This corona_4408 (HECoV-4408 or Human enteric coronavirus 4408) from cattle was shown to infect the intestine of humans (Zhang XM, Herbst W, Kousoulas KG, Storz J (1994). Biological and genetic characterization of a hemagglutinating coronavirus isolated from a diarrhoeic child. J Med Virol 44(2):152–61).In several cases, it was not possible to align the peptides with all sequences using standard algorithms. Then an alignment just with SARS-CoV-2 is shown.

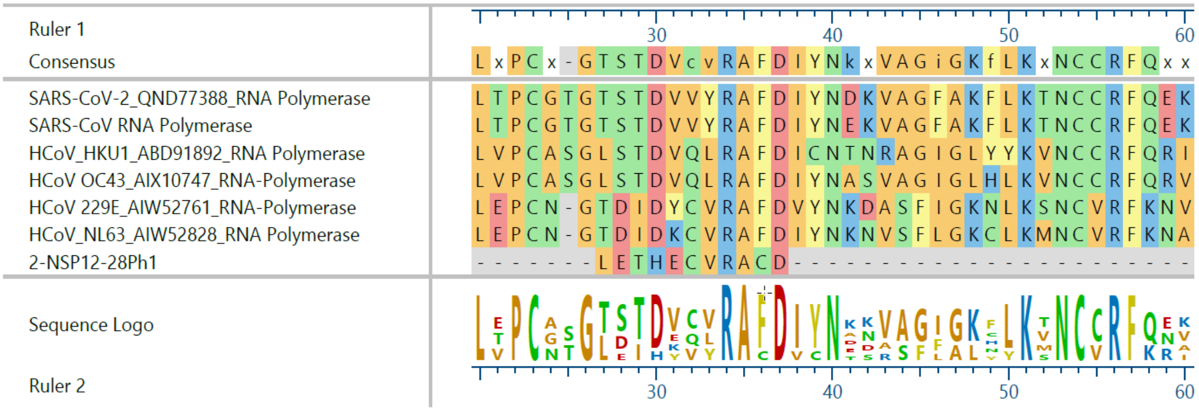

Figure S3-a 2-NSP12-28Ph1_RNA-pol-sequences

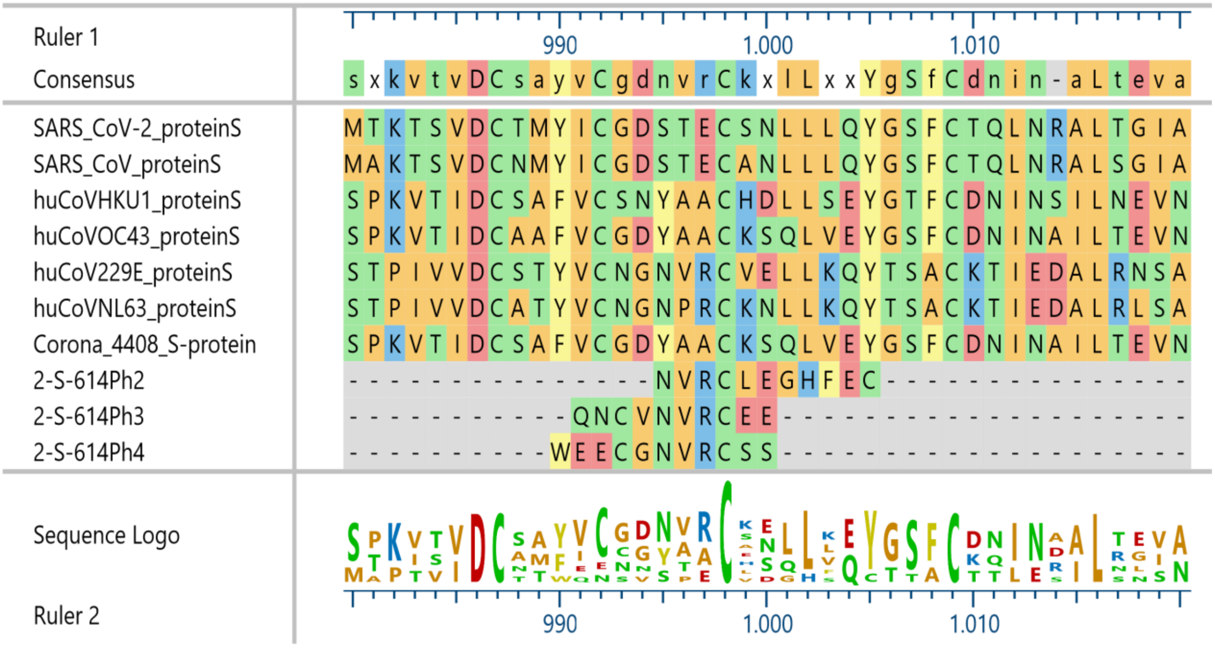

Figure S3-b 2-S-614all

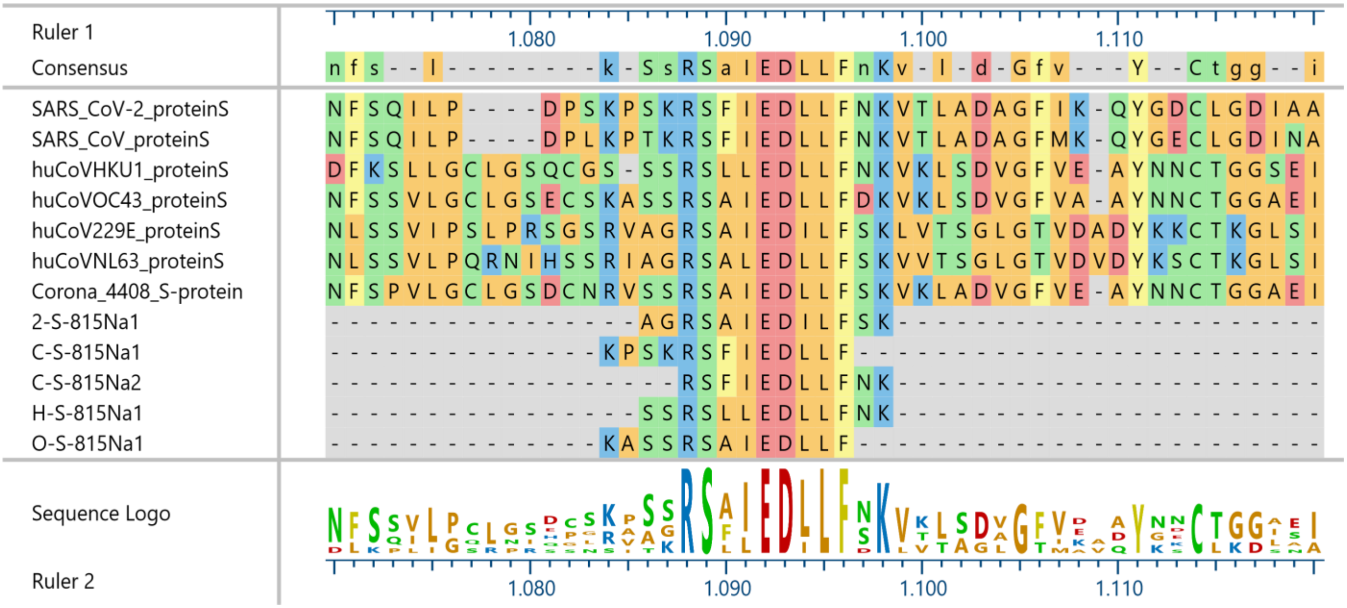

Figure S3-c all-815-s-protein

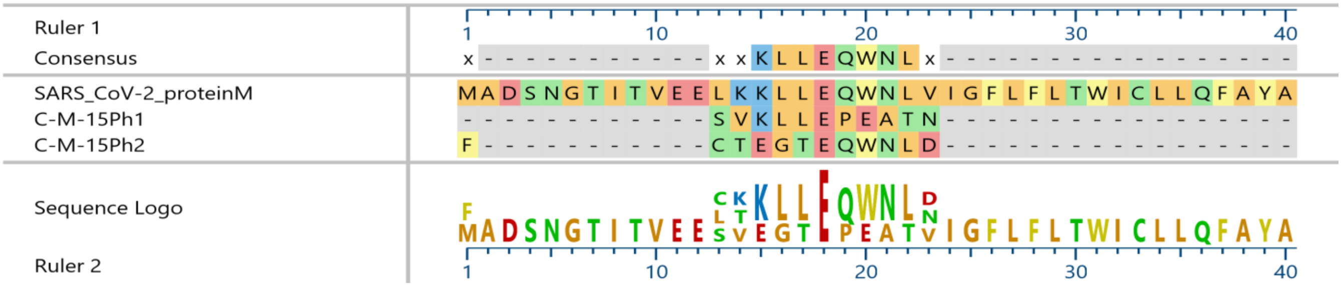

Figure S3-d C-M-15-all-partial

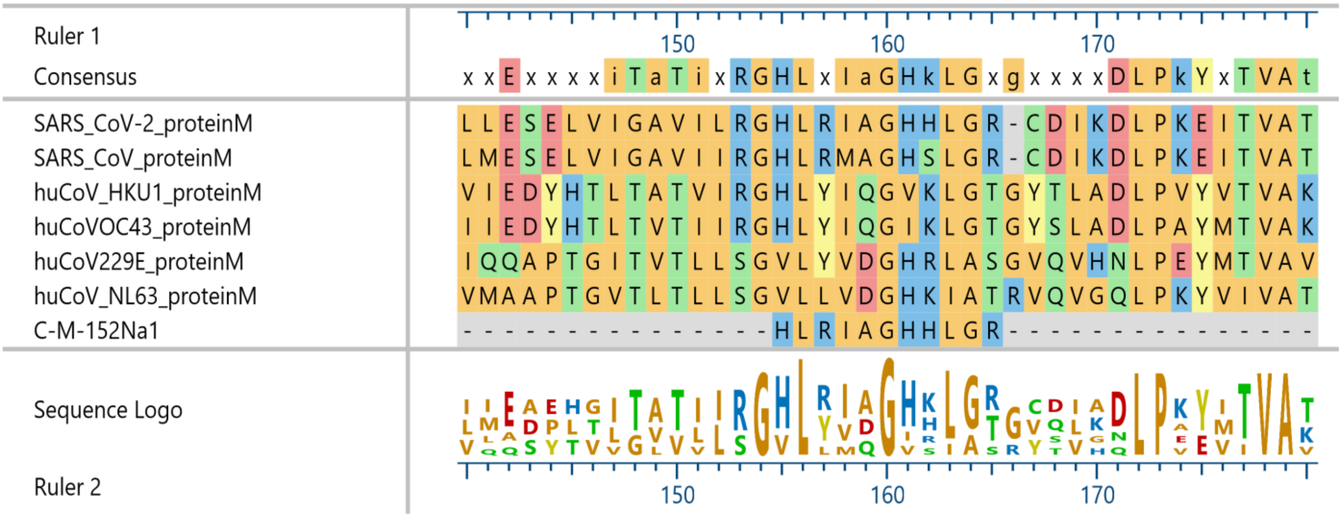

Figure S3-e C-M-152

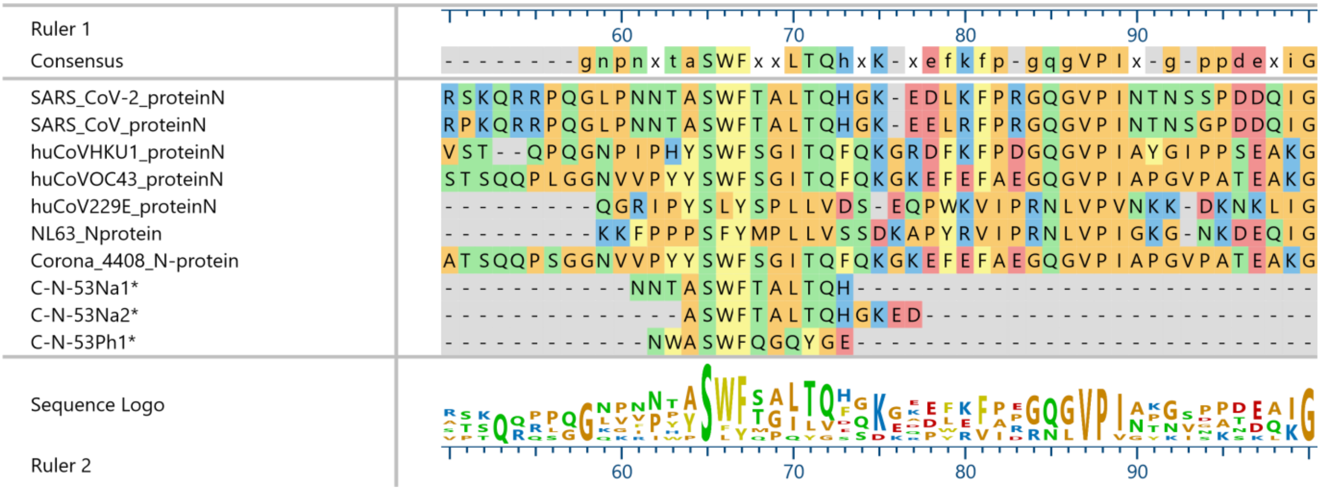

Figure S3-f C-N-53all

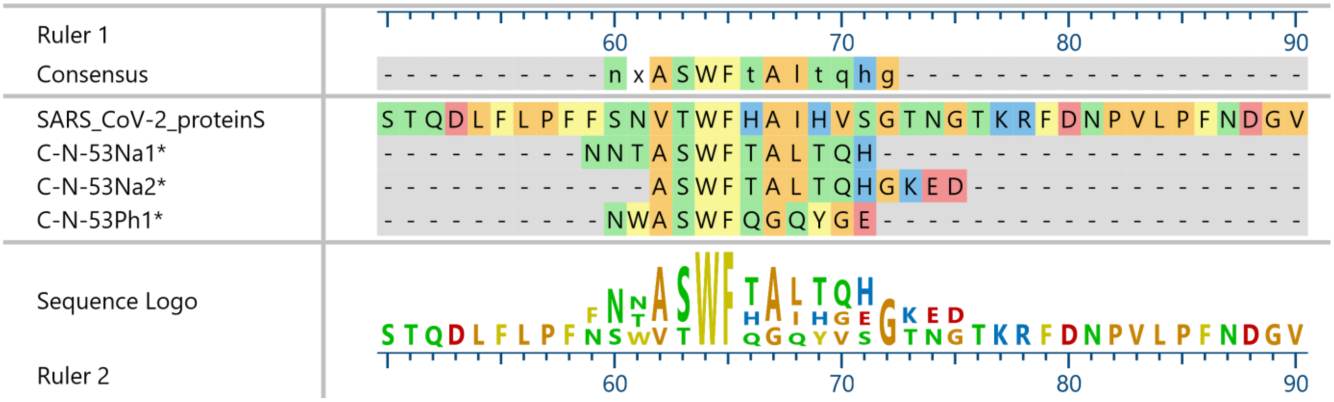

Figure S3-g C-N-53-S-protein

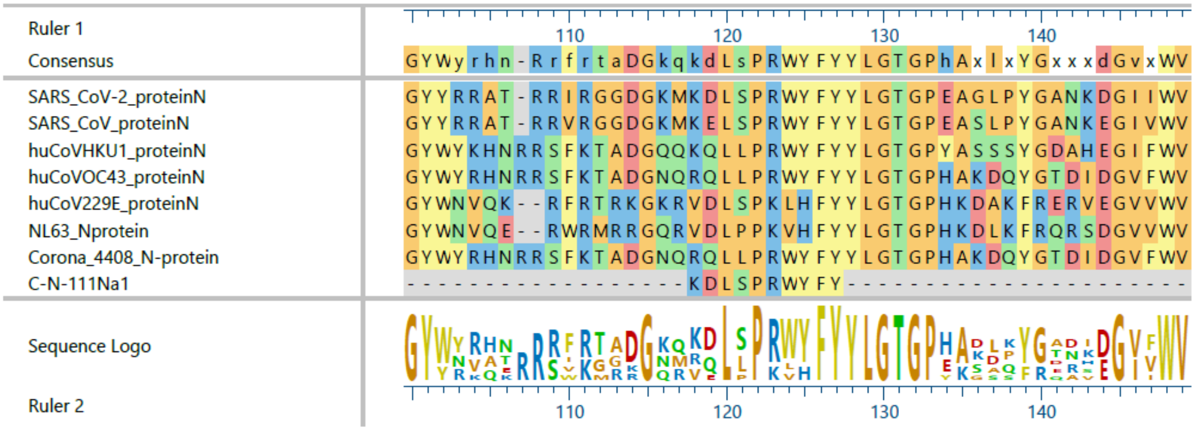

Figure S3-h C-N-111Na1

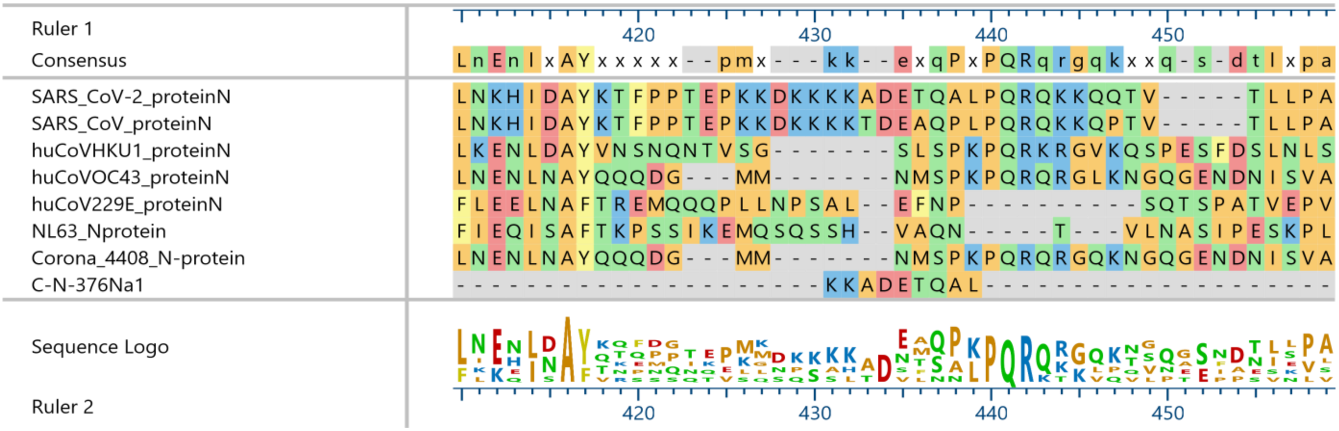

Figure S3-i C-N-376Na1

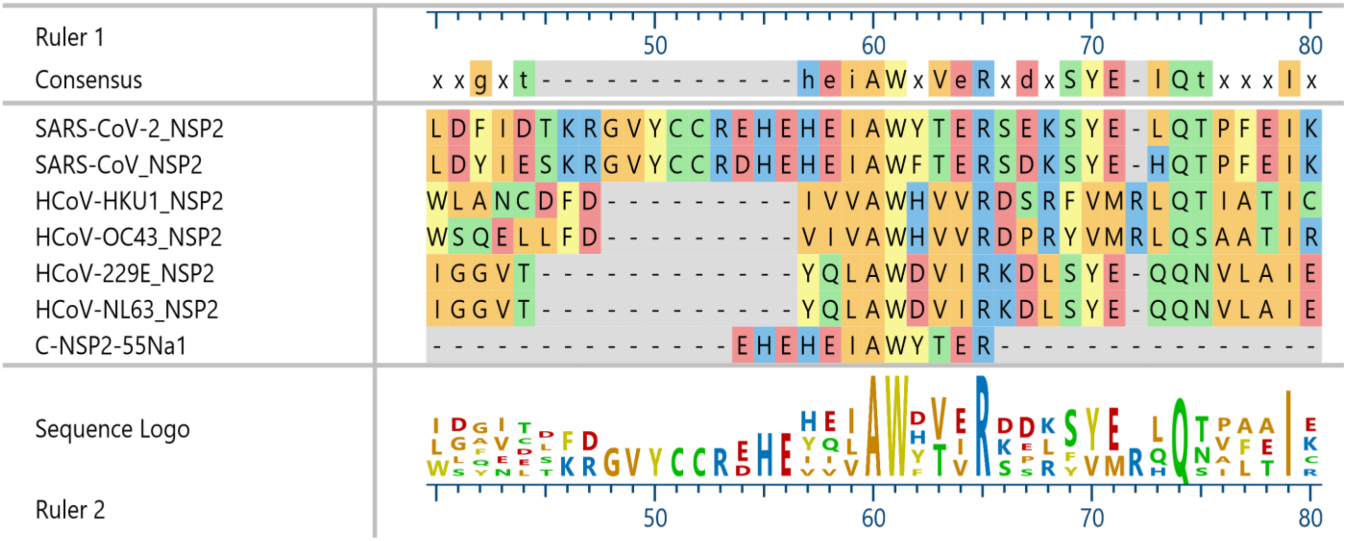

Figure S3-j C-NSP2-55Na1

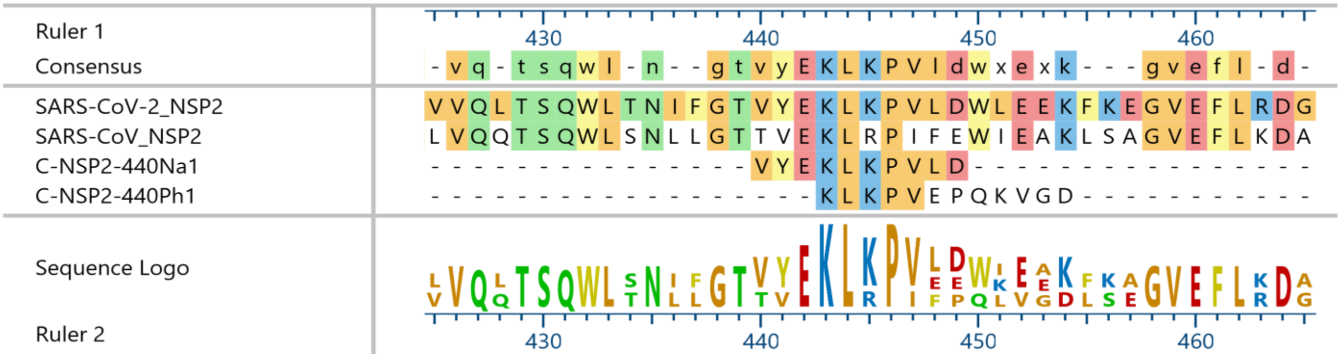

Figure S3-k C-NSP2-440all

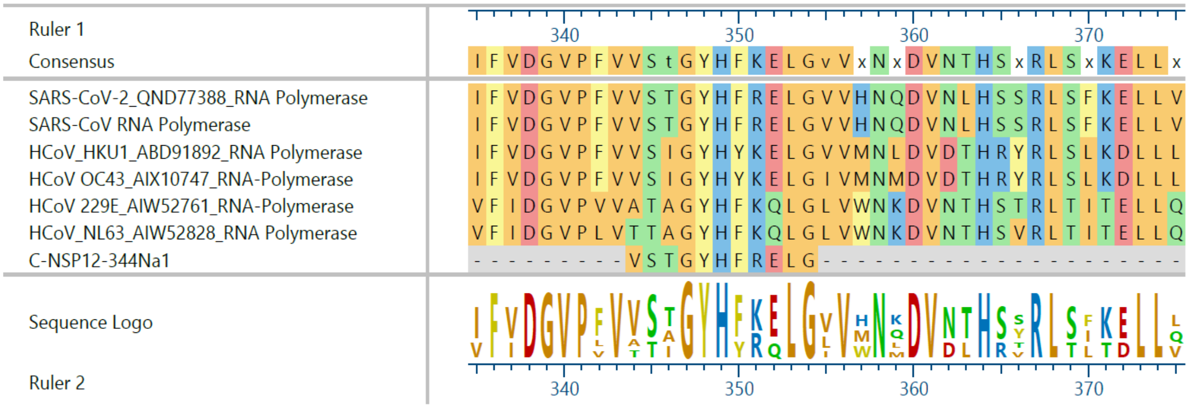

Figure S3-l C-NSP12-344Na1_RNA-pol-sequences

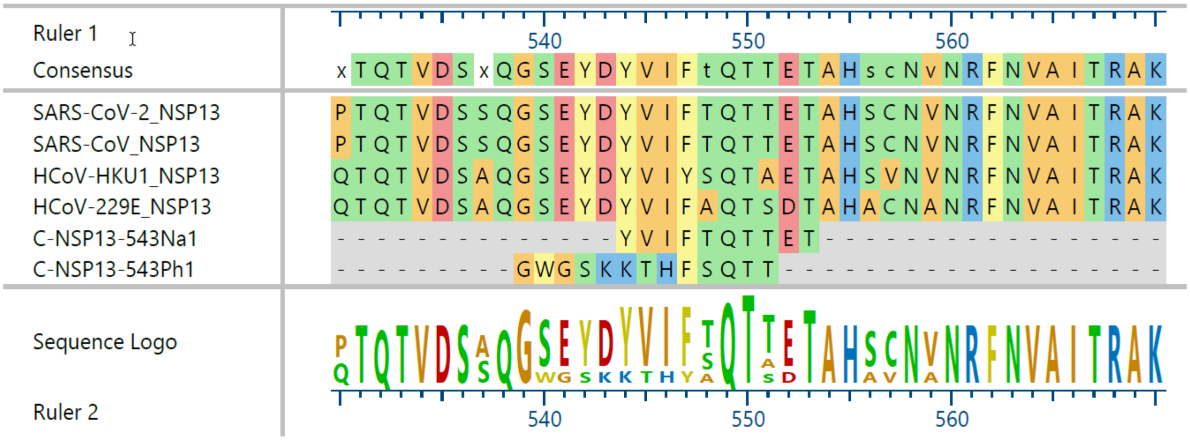

Figure S3-mC-NSP13_543all

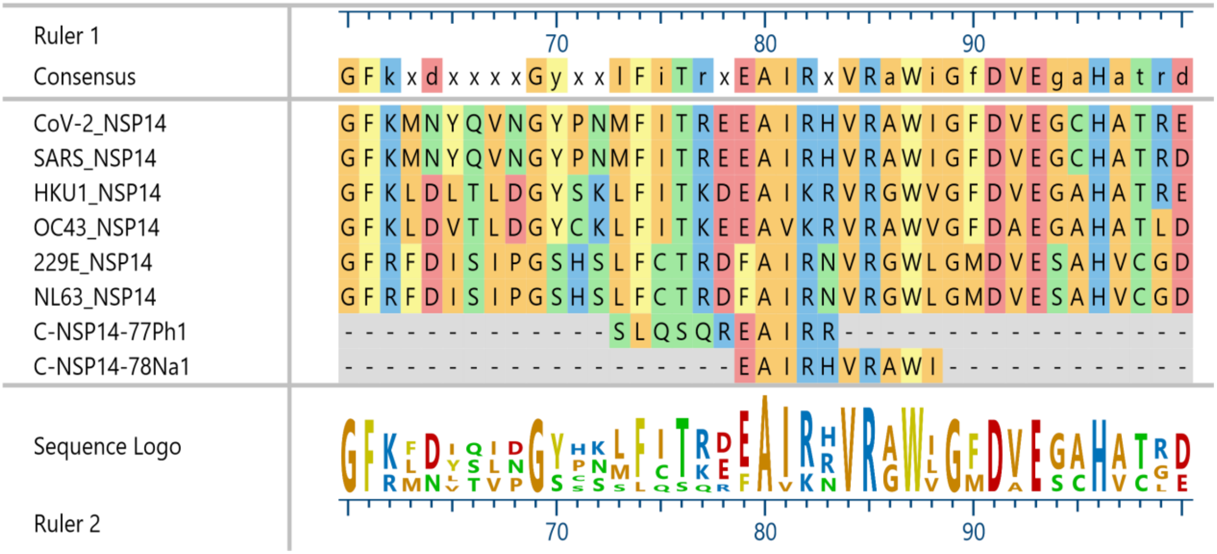

Figure S3-n C-NSP14-78all

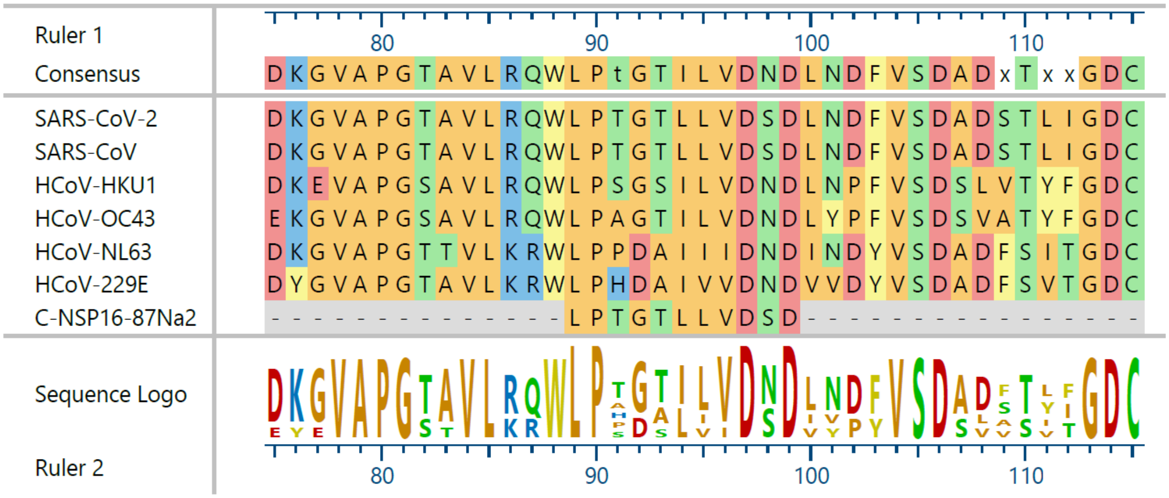

Figure S3-o C-NSP16-87Na2

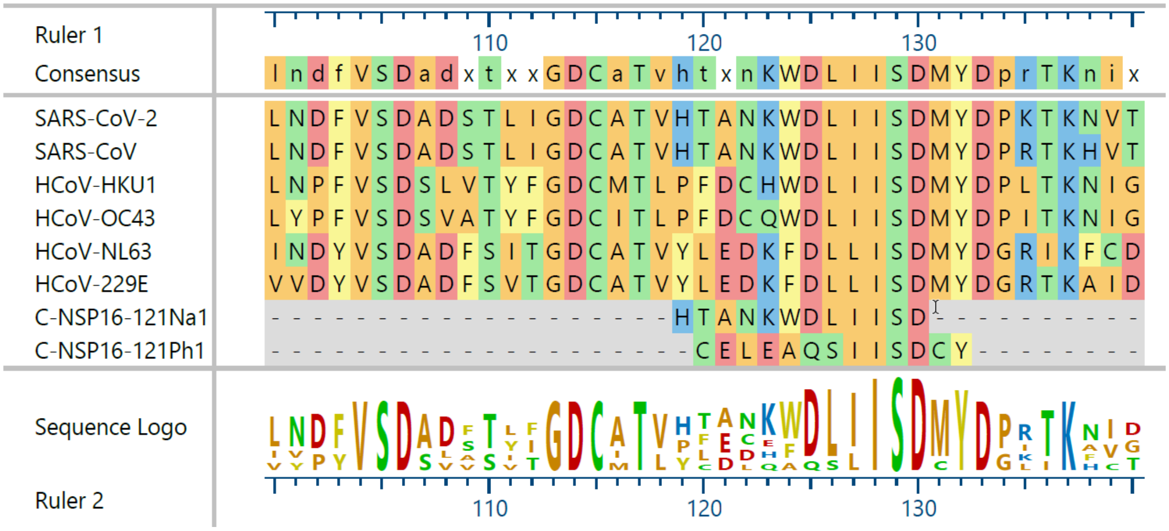

Figure S3-p C-NSP16-121all

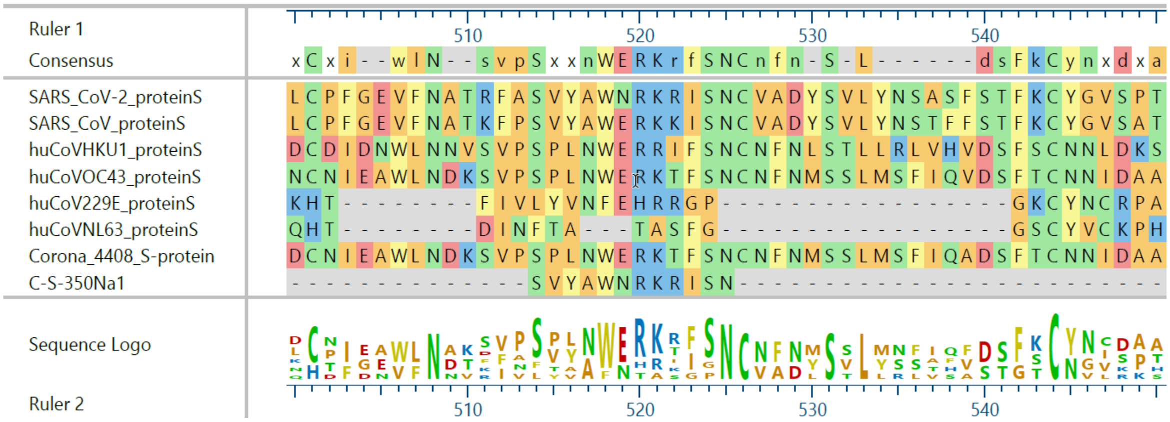

Figure S3-q C-S-350Na1

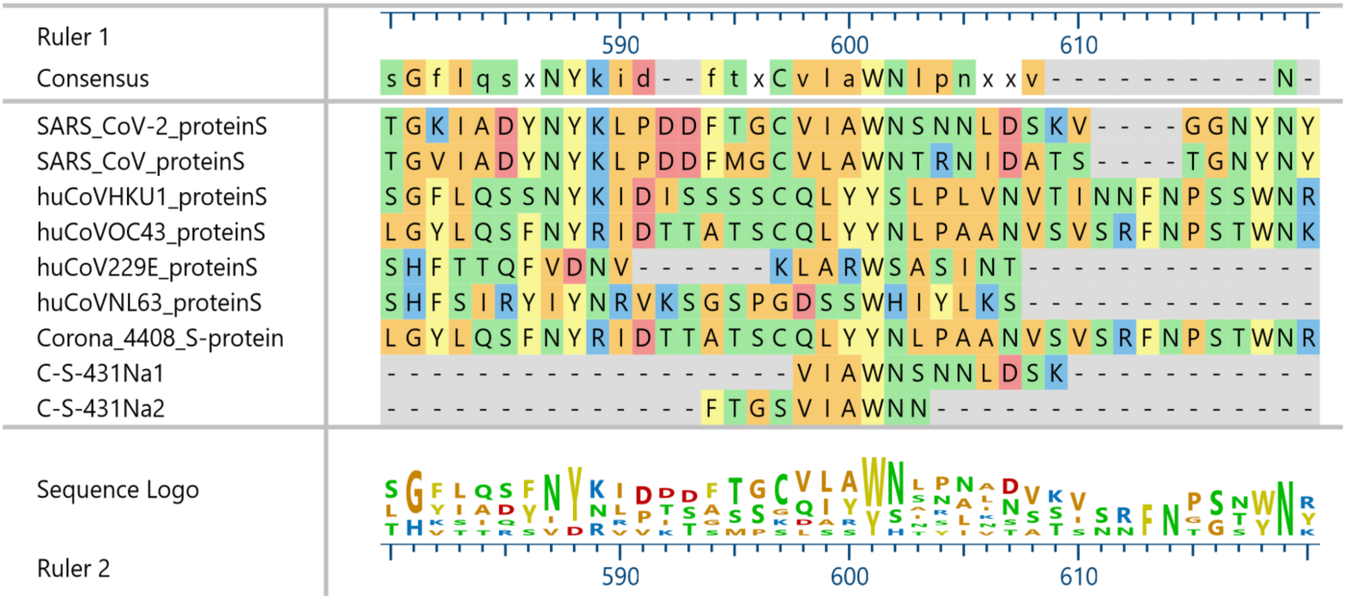

Figure S3-r C-S-431all

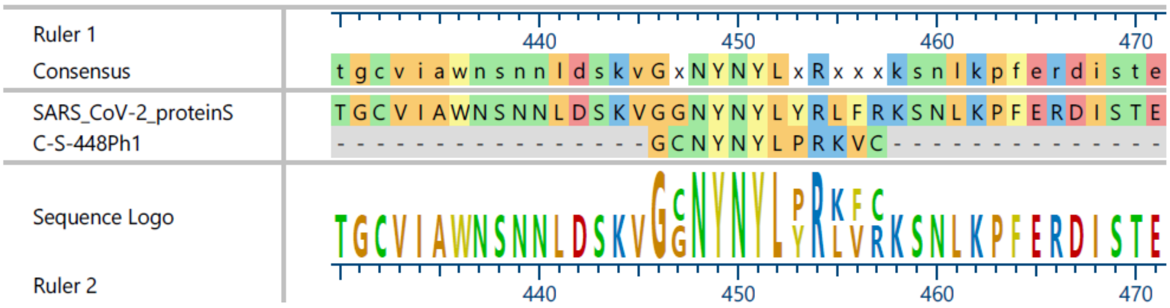

C-S-448Ph1

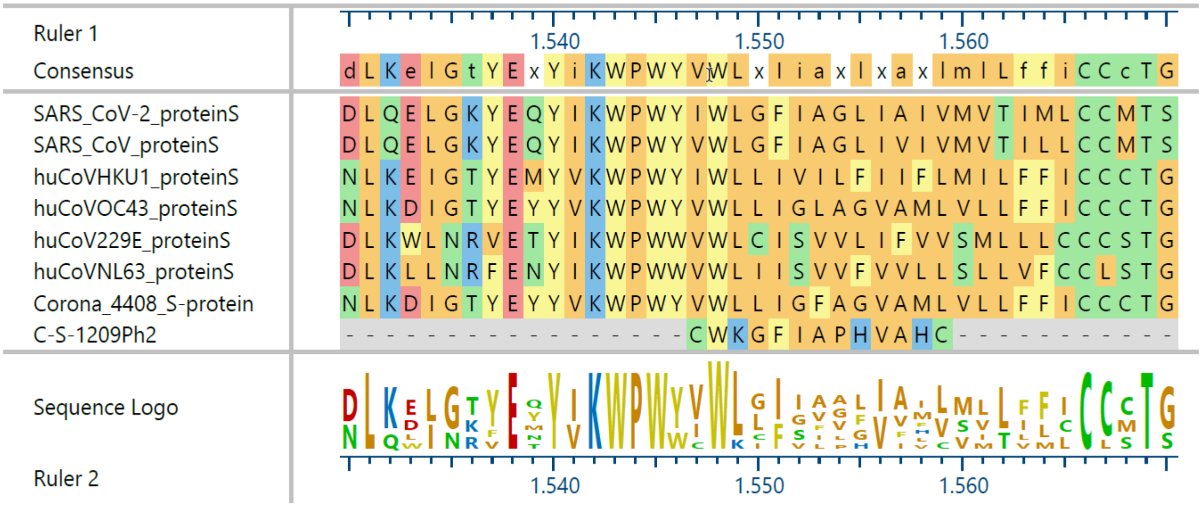

C-S-1209Ph2

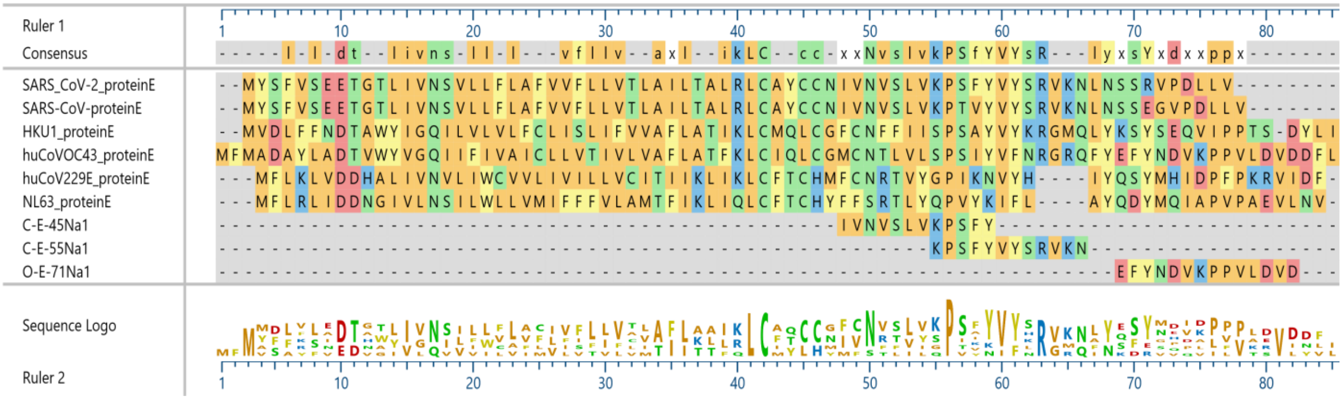

Figure S3-s E-proteins-sequences

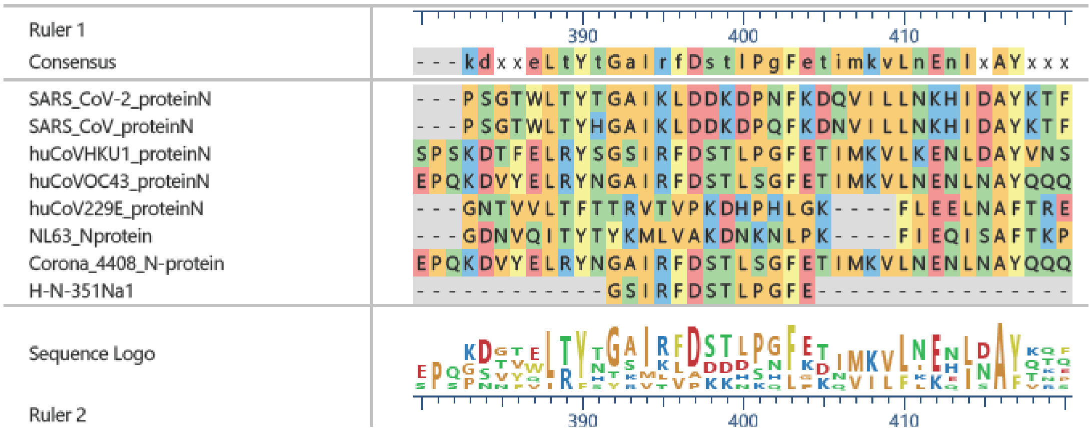

Figure S3-t H-N-351Na1

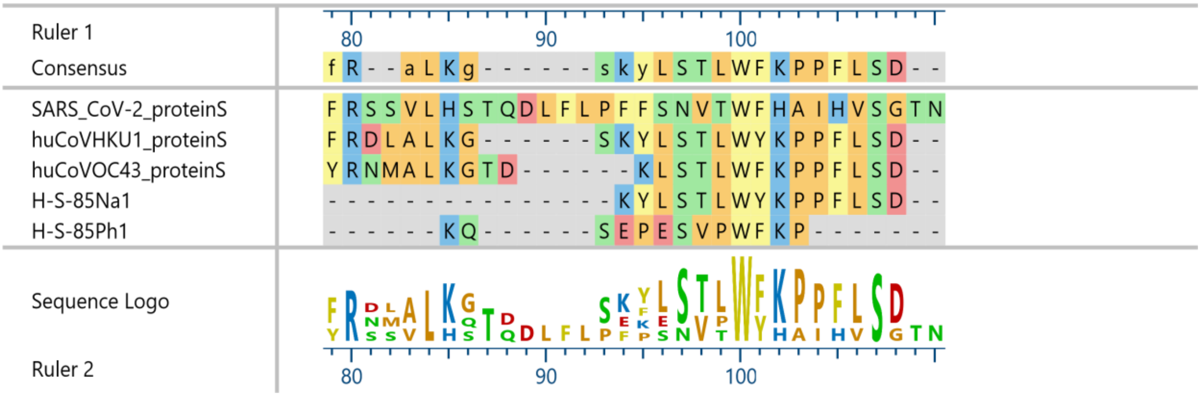

Figure S3-u H-S-85all

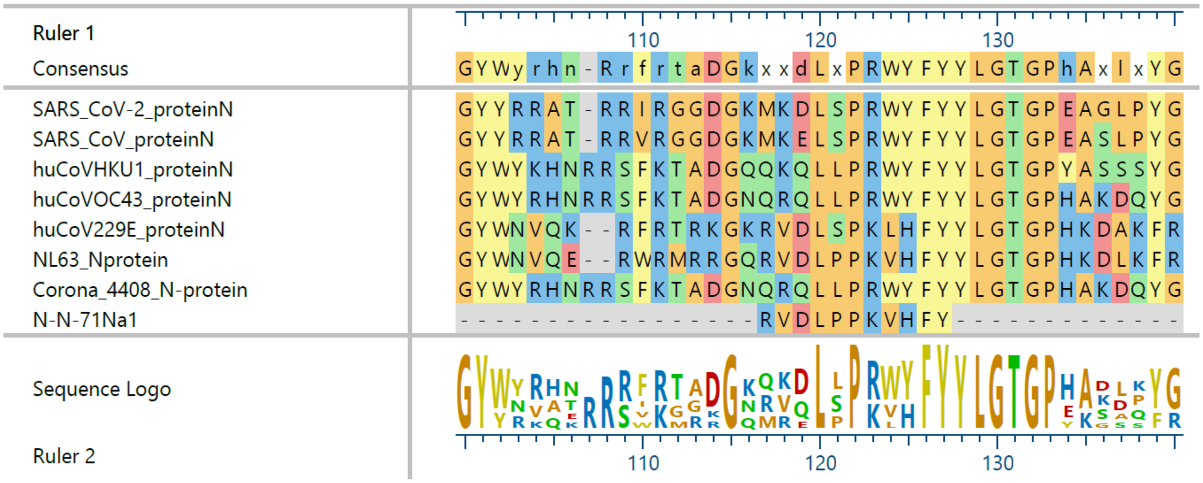

Figure S3-v N-N-71Na1

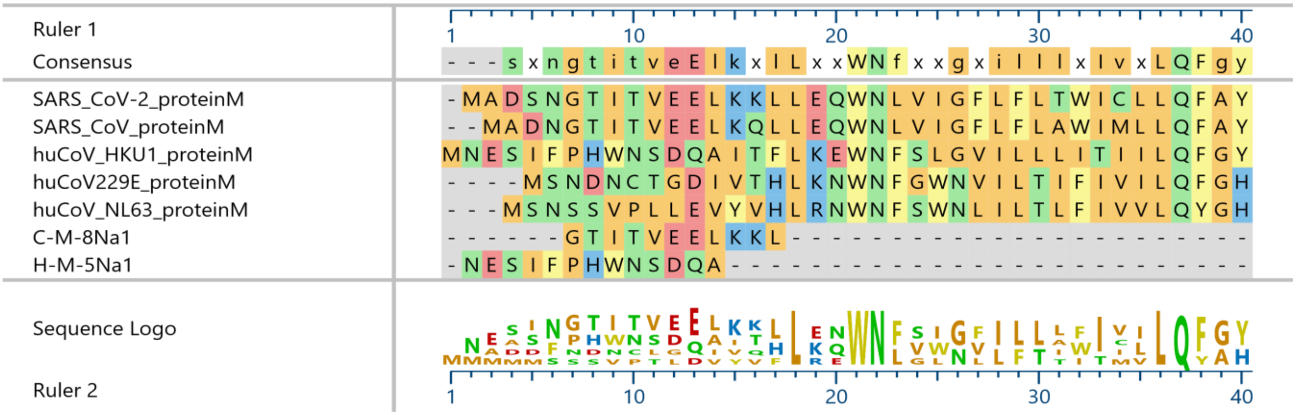

Figure S3-w N-term_epitopes_M-proteins

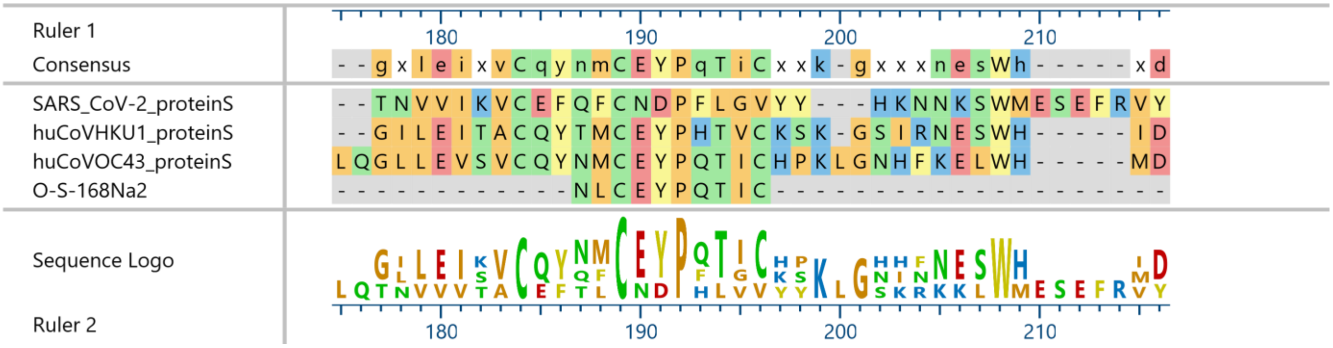

Figure S3-x O-S-168Na2

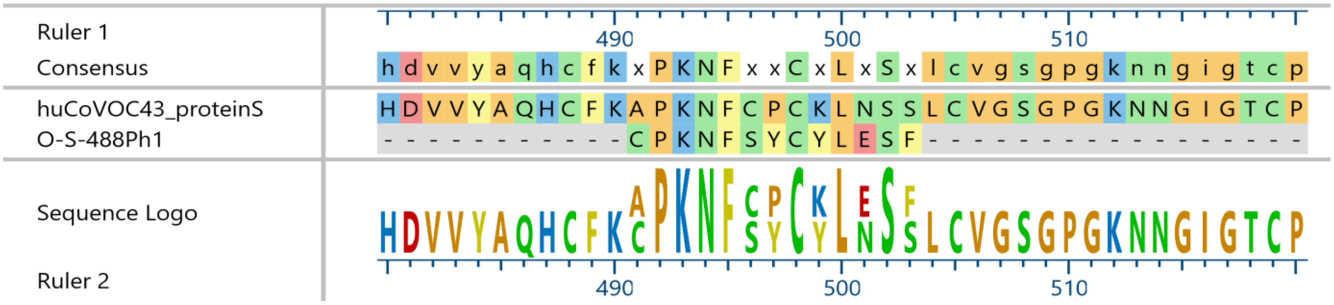

Figure S3-y O-S-488Ph1

S4. Heatmap

This diagram is a heatmap of intensities corresponding to Figure 2 in the article. Large variations of intensities between patients as well as epitope peptides make it more difficult to understand relevance of individual epitope peptides. Setup, patients and peptide epitopes are identical to Figure 2.

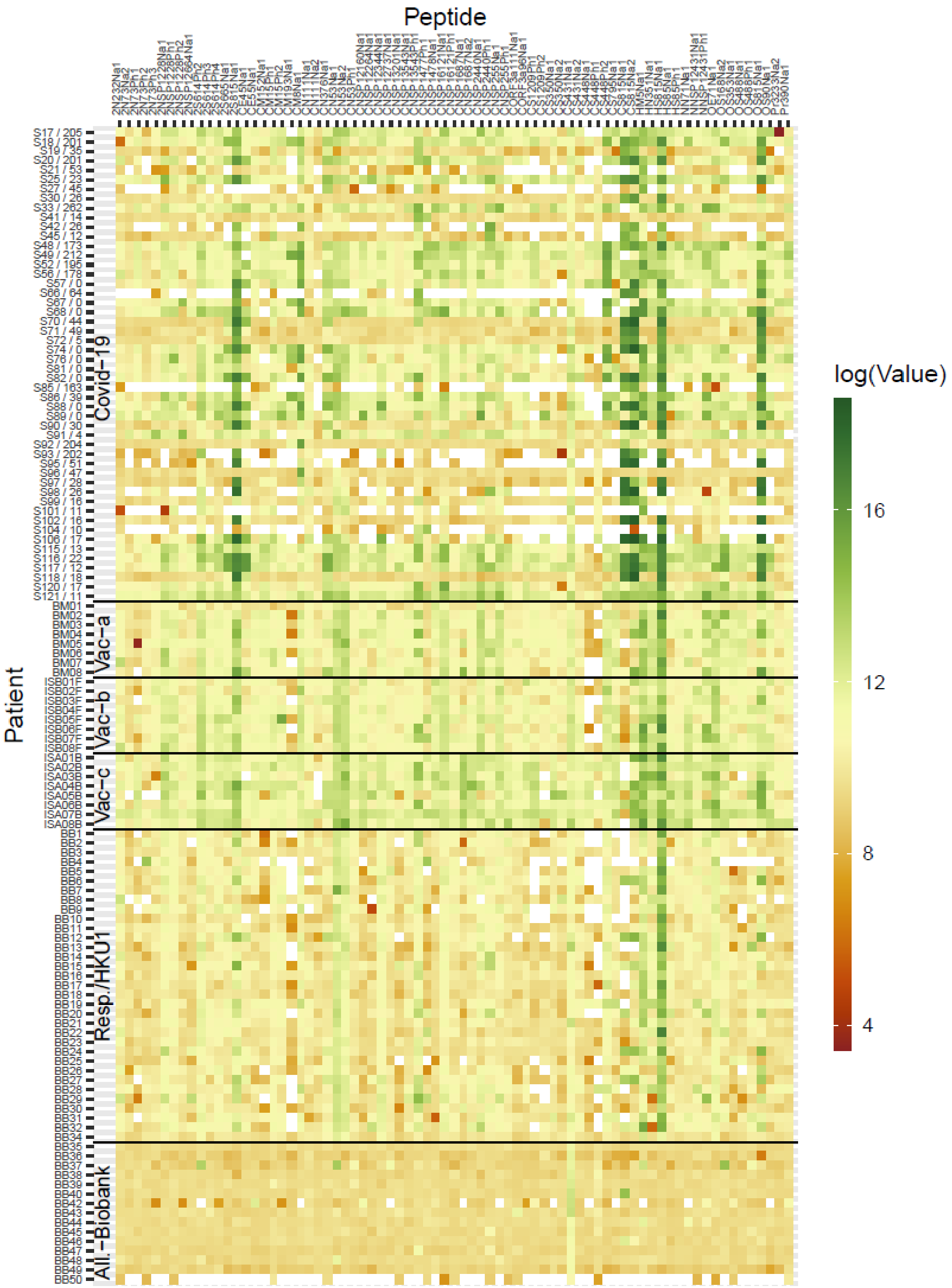

S5. Measurements of epitopes in the group of infectious respiratory disease patients

The following table summarizes antibody binding to epitopes from 104 patient sera suffering from respiratory disease and collected before the outbreak of the COVID-19 pandemia. Measurements were carried out with a small array focused on endemic corona virus strains. The patients with the strongest HKU-1 response were used for the measurements in Figure 2 and Figure 3 of the main article. Measurements were carried out in the same way as described above.

Submitted Manuscript: Template updated August 2023

Szardenings et.al. Supplement

	229E														CoV2		HKU1					NL63			OC43						
Patient	2N232Na1	2N73Na2	2N73Ph1	2N73Ph2	2N73Ph3	2NSP1228Na1	2NSP1228Ph1	2NSP1228Ph2	2NSP12664Na1	2S614Ph2	2S614Ph3	2S614Ph4	2S665Na1	2S815Na1	CE45Na1	CS815Na2	HM5Na1	HN351Na1	HS815Na1	HS85Na1	HS85Ph1	NN71Na1	NNSP12431Na1	NNSP12431Ph1	OE71Na1	OS168Na2	OS353Na1	OS488Na1	OS488Ph1	OS815Na1	OS90Na1
	N	N	N	N	N	RNA-POL				S	S	S	S	S	E	S	M	N	S	S	S	N	RNAPOL		E	S	S	S	S	S	S
1	0	0	0	0	0	0	0	0	0	1	0	0	0	0	0	0	1	0	0	0	0	0	0	0	0	0	0	0	0	0	0
2	0	0	0	0	0	0	0	0	0	0	1	0	n.d.	0	0	0	1	0	1	0	0	0	0	0	0	0	0	0	0	0	0
3	n.d.	0	0	0	0	0	0	0	n.d.	1	0	0	0	0	0	0	1	0	1	0	0	0	0	1	1	1	0	1	0	1	0
4	0	n.d.	0	0	0	0	0	0	0	0	0	0	0	0	0	0	1	0	1	0	0	0	0	0	0	0	0	0	0	0	0
5	0	0	0	0	0	0	0	0	n.d.	1	0	0	0	0	0	0	1	1	1	0	0	0	0	0	1	0	0	1	1	0	0
6	0	n.d.	0	0	0	0	0	0	0	1	1	0	0	1	0	0	1	0	1	0	0	1	1	1	0	0	1	1	0	1	0
7	0	0	0	0	0	0	0	0	0	1	0	0	0	0	0	0	0	0	1	0	0	0	0	0	0	0	0	0	0	0	0
8	0	0	1	0	1	0	1	1	0	1	1	1	0	1	0	1	1	1	1	1	1	1	1	1	0	1	0	1	1	1	1
9	0	0	0	0	n.d.	0	0	0	n.d.	1	0	1	0	0	0	0	1	1	1	0	0	1	1	1	1	0	0	1	0	1	0
10	0	0	0	0	0	0	0	0	0	0	1	0	0	0	0	0	0	0	1	0	0	0	0	0	0	0	0	0	0	0	0
11	n.d.	0	0	0	0	0	0	n.d.	0	1	0	n.d.	0	n.d.	0	0	0	n.d.	1	n.d.	0	0	0	0	0	0	0	1	0	0	0
12	0	0	0	0	0	0	0	0	0	1	1	0	0	0	0	0	1	0	1	0	0	1	1	1	0	0	0	1	0	1	0
13	0	0	0	0	0	0	0	n.d.	n.d.	1	0	0	0	1	0	1	1	0	1	0	0	0	0	0	0	0	0	1	0	1	0
14	0	0	0	0	0	0	0	0	0	1	0	0	0	0	0	0	1	0	1	0	0	1	0	0	0	0	0	0	0	0	0
15	0	0	0	0	0	0	0	0	0	1	1	0	0	0	0	n.d.	1	0	0	n.d.	0	0	0	0	0	0	0	1	0	0	0
16	0	n.d.	0	n.d.	n.d.	0	0	0	0	1	0	0	0	0	n.d.	0	1	0	1	0	0	0	0	1	0	0	n.d.	0	0	0	0
17	0	0	0	0	0	0	0	0	n.d.	0	0	0	0	0	0	0	0	0	1	0	0	0	0	0	0	0	0	0	0	0	0
18	0	n.d.	0	0	0	0	0	0	0	1	0	0	0	0	0	n.d.	0	0	1	0	0	0	0	0	0	1	0	0	0	0	0
19	0	0	0	0	0	0	0	n.d.	0	0	0	0	0	n.d.	0	n.d.	0	0	1	0	0	0	0	0	0	0	0	1	0	0	0
20	n.d.	n.d.	n.d.	n.d.	n.d.	n.d.	1	n.d.	n.d.	1	0	0	0	0	1	n.d.	1	1	1	0	0	1	0	0	1	0	0	1	0	0	0
21	0	n.d.	0	0	0	n.d.	0	n.d.	n.d.	0	0	0	0	n.d.	0	1	1	0	1	1	0	0	0	0	0	0	0	0	0	1	0
22	0	0	0	0	0	0	0	0	0	0	0	0	0	0	0	0	0	0	1	0	0	0	0	0	0	0	0	0	0	0	0
23	0	0	0	0	0	0	0	0	0	0	0	0	0	0	0	0	0	0	0	0	0	0	0	0	0	0	0	0	0	0	0
24	0	0	0	0	0	n.d.	0	0	0	0	0	0	0	0	0	0	0	0	0	0	0	0	0	0	0	0	0	0	0	0	0
25	0	0	0	0	0	0	0	0	0	1	1	0	0	0	0	0	1	0	1	0	0	0	0	1	0	0	0	1	0	1	0
26	0	0	0	0	0	0	0	0	0	1	1	0	0	0	0	0	1	0	1	0	0	0	0	0	0	1	0	1	0	0	0
27	0	0	0	0	0	0	0	0	0	0	0	0	0	0	0	0	1	0	1	0	0	0	0	0	0	0	0	0	0	0	0
28	0	0	0	0	0	0	0	0	0	1	0	0	0	0	0	0	1	0	0	0	0	0	0	0	0	1	0	0	0	0	0
29	0	n.d.	0	n.d.	0	0	0	n.d.	0	1	1	0	n.d.	0	0	0	0	0	1	0	0	0	0	0	0	0	0	0	0	0	0
30	0	n.d.	0	0	0	n.d.	0	0	n.d.	1	0	0	0	1	0	1	0	0	1	0	0	0	0	0	0	0	0	0	0	1	0
31	0	0	0	n.d.	0	0	0	0	n.d.	1	1	0	0	1	0	0	1	0	0	n.d.	0	0	0	0	0	0	0	1	0	0	0
32	0	n.d.	0	n.d.	0	0	0	0	n.d.	1	0	0	0	0	0	0	1	0	1	n.d.	0	1	0	0	0	0	n.d.	1	0	1	0
33	0	0	0	0	0	0	0	0	0	0	0	0	0	0	0	0	1	0	1	0	0	1	0	0	0	0	0	0	0	0	0
34	0	0	0	0	0	0	0	0	0	0	0	0	0	0	0	0	0	0	1	1	0	0	0	0	0	0	0	0	0	0	0
35	0	0	0	0	0	0	0	0	0	0	0	0	0	0	n.d.	0	0	0	1	0	0	0	0	0	0	0	0	0	0	0	0
36	0	n.d.	0	0	0	n.d.	0	0	n.d.	0	0	0	n.d.	1	0	1	1	0	1	0	0	0	0	0	0	0	0	0	0	1	n.d.
37	0	0	0	0	0	0	0	0	0	1	1	0	0	0	n.d.	0	0	0	0	0	0	0	0	0	0	0	0	0	0	0	n.d.
38	0	0	0	0	0	0	0	0	0	0	0	0	0	0	0	0	0	0	1	0	0	0	0	0	0	0	0	0	0	0	0
39	0	0	0	0	0	0	0	0	0	0	0	0	0	0	0	0	1	0	0	0	0	0	0	0	0	0	0	0	0	0	0
40	n.d.	n.d.	0	n.d.	0	n.d.	0	0	n.d.	0	0	0	0	0	n.d.	0	1	0	1	0	0	0	1	0	0	0	0	1	0	0	n.d.
41	1	n.d.	0	n.d.	0	1	0	0	1	1	1	0	0	1	0	1	1	0	1	0	0	0	0	1	0	1	0	0	0	1	0
42	0	n.d.	0	n.d.	0	0	0	n.d.	n.d.	1	1	0	0	0	0	0	1	0	1	0	0	0	0	0	0	1	0	1	0	0	0
43	0	0	0	n.d.	0	0	0	0	n.d.	1	0	0	0	0	n.d.	0	1	0	1	0	0	0	0	0	0	0	0	1	0	0	n.d.
44	0	0	0	0	0	0	1	0	0	1	1	0	0	0	0	0	1	0	1	0	0	0	0	0	0	0	0	0	0	1	0
45	0	0	0	0	0	0	0	0	n.d.	1	1	0	0	0	n.d.	0	1	0	1	0	0	0	0	0	0	0	0	0	0	0	n.d.
46	0	0	0	0	0	0	0	0	0	1	1	0	0	0	n.d.	0	1	0	1	1	0	0	0	0	0	0	0	1	0	0	n.d.
47	0	0	0	0	0	0	1	0	0	1	1	1	0	0	0	0	1	0	1	0	0	0	0	0	0	0	0	0	0	1	0
48	0	1	0	n.d.	0	1	1	0	n.d.	1	1	0	0	0	n.d.	0	1	0	1	0	0	0	1	0	0	1	0	1	0	1	n.d.
49	n.d.	0	0	0	0	0	0	0	n.d.	0	0	0	0	0	0	0	0	0	1	0	0	0	0	1	0	0	0	1	0	0	0
50	0	0	0	0	0	0	0	0	0	0	0	0	0	0	0	0	1	0	1	0	0	0	0	0	0	0	0	1	0	0	0
51	0	0	0	0	0	0	0	0	0	1	0	0	0	0	n.d.	0	1	0	1	1	0	1	0	0	1	0	0	1	1	0	n.d.
52	n.d.	n.d.	0	n.d.	n.d.	n.d.	0	0	n.d.	0	0	n.d.	0	0	0	n.d.	1	0	1	0	1	0	0	0	0	0	0	1	0	0	0
53	0	0	0	0	0	0	0	0	0	1	0	0	0	0	n.d.	0	0	0	1	0	1	0	0	1	0	0	0	1	0	0	n.d.
54	n.d.	0	0	0	0	0	0	0	n.d.	1	1	0	0	1	n.d.	1	1	0	1	0	0	0	0	0	1	0	0	1	0	1	1
55	n.d.	0	0	0	0	0	0	n.d.	0	0	0	0	0	0	n.d.	0	0	0	0	n.d.	0	0	0	0	0	0	0	0	0	0	n.d.
56	0	0	0	0	0	0	0	0	0	1	1	0	0	0	0	0	0	0	1	1	0	0	0	0	0	0	0	0	0	0	0
57	0	0	0	0	0	0	0	0	0	0	0	0	0	0	0	1	1	0	1	0	0	0	0	0	0	0	0	0	0	0	0
58	0	n.d.	0	0	0	0	0	0	n.d.	0	1	0	0	0	0	0	0	0	1	0	0	0	0	1	0	0	0	1	0	0	0
59	0	0	0	0	0	0	1	0	0	1	0	0	0	1	n.d.	0	1	0	1	0	0	1	0	0	0	0	0	1	0	1	n.d.
60	0	0	0	0	0	0	0	0	0	1	0	0	0	0	0	0	0	0	0	0	0	1	0	0	0	0	0	0	0	0	0
61	0	0	0	0	0	0	0	0	0	1	0	0	0	0	n.d.	0	1	1	1	0	0	0	0	0	0	0	0	1	0	1	n.d.
62	0	0	0	0	1	0	0	0	0	1	1	0	0	0	0	0	0	0	0	0	0	0	0	0	0	0	0	0	0	0	0
63	0	0	0	0	0	0	0	0	0	1	1	0	0	0	0	n.d.	1	0	0	0	0	0	0	0	0	0	0	0	0	0	0
64	0	0	0	0	0	0	0	0	0	1	1	0	0	0	0	0	1	0	1	0	0	0	0	1	0	0	0	0	0	0	0
65	0	0	0	n.d.	0	0	0	0	n.d.	1	1	0	0	1	0	1	1	0	1	0	0	0	0	0	0	0	n.d.	0	0	1	0
66	0	0	0	0	0	0	0	0	0	0	0	0	0	0	0	0	0	0	0	0	0	0	0	1	0	0	0	0	0	0	0
67	n.d.	n.d.	n.d.	n.d.	n.d.	n.d.	0	n.d.	n.d.	0	0	0	n.d.	n.d.	0	n.d.	n.d.	0	0	n.d.	0	0	0	0	0	0	n.d.	0	0	0	0
68	0	0	0	0	0	0	0	0	0	0	0	0	0	0	0	0	0	0	0	0	0	0	0	0	0	0	0	0	0	0	0
69	0	0	0	0	0	0	0	0	0	0	0	0	0	0	0	0	1	0	1	0	0	0	0	0	0	0	0	0	0	0	0
70	0	0	0	0	0	0	0	0	0	0	0	0	0	0	0	0	0	0	1	0	0	0	0	0	0	0	0	0	0	0	0
71	0	0	0	0	0	n.d.	0	0	n.d.	0	0	0	n.d.	0	0	0	0	0	1	n.d.	0	0	0	0	0	0	0	0	0	1	0
72	0	0	0	n.d.	0	0	0	0	n.d.	1	0	0	0	n.d.	0	0	0	0	0	0	0	0	0	0	0	0	0	1	0	0	0
73	0	0	0	0	0	0	1	0	0	1	1	0	n.d.	0	0	0	1	0	1	0	0	0	0	1	0	0	0	1	0	0	0
74	0	0	0	n.d.	n.d.	0	0	0	n.d.	1	0	0	0	0	0	0	0	0	0	0	0	1	0	0	0	0	0	0	0	0	0
75	0	0	0	n.d.	n.d.	n.d.	0	0	n.d.	0	0	0	n.d.	n.d.	0	n.d.	0	1	0	n.d.	0	0	0	0	0	0	0	0	0	n.d.	n.d.
76	0	0	0	0	0	0	0	0	0	1	0	0	0	0	0	0	1	0	0	0	0	0	0	0	0	0	0	0	0	0	0
77	0	0	0	0	0	0	0	0	0	1	1	0	0	0	0	0	0	0	0	0	0	0	0	1	0	0	0	0	0	0	0
78	0	0	0	0	0	0	0	0	0	1	0	0	0	0	0	0	0	0	1	0	0	0	0	0	0	0	0	0	0	0	0
79	0	0	0	0	0	0	0	0	0	1	0	0	0	0	0	0	0	0	0	0	0	0	0	0	0	0	0	0	0	0	0
80	0	n.d.	0	0	0	0	0	0	0	0	0	0	0	0	0	0	1	0	0	0	0	0	0	0	0	0	0	0	0	0	0
81	n.d.	n.d.	0	n.d.	n.d.	n.d.	0	n.d.	n.d.	0	0	0	n.d.	0	0	0	0	0	1	0	0	0	0	0	0	0	0	0	0	1	0
82	0	0	0	0	0	0	0	0	0	0	0	0	0	1	0	0	1	0	0	0	0	0	0	0	0	0	0	1	0	0	0
83	0	0	0	0	n.d.	0	0	n.d.	0	1	0	0	0	0	0	n.d.	1	1	1	0	0	0	1	0	0	0	0	1	0	0	0
84	0	n.d.	0	0	0	0	0	0	n.d.	1	0	0	0	0	0	0	1	0	1	0	0	1	0	0	0	0	0	1	0	0	0
85	0	0	0	0	0	0	0	0	0	0	0	0	0	0	0	0	0	0	0	0	0	0	0	1	0	0	0	0	0	0	0
86	n.d.	n.d.	0	n.d.	n.d.	0	0	n.d.	n.d.	0	0	0	0	0	0	0	1	0	1	1	0	1	0	1	0	0	0	1	0	1	0
87	0	0	0	0	0	0	0	0	0	0	0	0	0	0	0	0	0	0	1	0	0	1	0	0	0	0	0	0	0	0	0
88	0	n.d.	0	n.d.	n.d.	0	0	n.d.	n.d.	0	0	0	n.d.	n.d.	0	n.d.	1	0	1	0	0	1	1	0	0	0	0	0	0	n.d.	0
89	0	0	0	0	0	0	0	n.d.	0	1	0	0	0	0	0	0	0	0	1	0	0	0	0	0	0	0	0	0	0	0	n.d.
90	0	0	0	0	0	0	0	0	0	1	0	0	0	0	0	0	1	0	1	0	0	0	0	0	0	1	0	1	0	0	0
91	0	0	0	0	0	0	0	0	0	1	1	0	0	0	0	0	1	0	1	0	0	0	0	0	0	0	0	0	0	0	0
92	0	0	0	0	0	0	0	0	0	1	0	0	0	0	0	0	1	0	1	0	0	1	0	0	0	0	0	1	0	0	0
93	0	0	0	0	0	0	0	0	0	0	0	0	0	0	0	0	1	0	1	0	0	0	0	0	0	0	0	0	0	0	0
94	0	0	0	0	0	0	0	0	0	1	1	0	0	1	0	1	1	0	1	0	0	0	0	0	1	0	0	0	0	1	0
95	0	0	0	0	0	0	0	0	0	0	0	0	0	0	0	0	0	0	0	0	0	0	0	0	0	0	0	0	0	0	0
96	0	0	0	0	0	0	0	0	0	0	0	0	0	0	0	0	0	0	1	1	0	1	0	0	0	0	0	0	0	0	0
97	0	0	0	0	0	0	1	0	0	1	1	0	n.d.	0	0	0	1	0	1	0	0	0	0	1	0	0	0	0	0	0	0
98	0	n.d.	0	n.d.	0	0	0	0	n.d.	1	1	0	0	0	0	0	1	0	1	n.d.	0	0	0	0	0	0	0	0	0	0	0
99	0	0	n.d.	n.d.	n.d.	n.d.	0	0	n.d.	1	1	0	n.d.	0	0	0	1	0	1	0	0	0	0	0	0	0	0	0	0	0	0
100	0	0	0	0	0	0	0	0	0	0	0	0	0	0	0	0	1	0	1	0	0	0	0	0	0	0	0	1	0	0	0
101	0	0	0	0	0	1	0	0	0	0	1	0	0	0	0	1	1	0	1	0	0	0	0	1	0	0	0	0	0	1	0
102	0	n.d.	1	n.d.	0	0	0	0	0	1	1	0	0	0	0	0	1	0	1	0	0	0	0	0	0	0	0	0	0	0	0
103	0	0	1	0	0	0	0	0	0	1	0	0	0	0	0	0	1	0	1	0	0	0	0	0	0	1	0	1	0	0	0
104	0	0	0	0	0	0	0	0	0	0	0	0	0	1	0	0	1	0	0	0	0	0	1	0	0	0	0	1	0	1	0
